# Risk factors and prediction model for lymph node metastasis posterior to the right recurrent laryngeal nerve in papillary thyroid carcinoma

**DOI:** 10.1186/s12957-025-04012-9

**Published:** 2025-10-14

**Authors:** Xiaowen Fang, Jihao Qin, Chenxi Liang, Siyu Li, Xueyu Zeng, Zhu Chen, Jie-Hua Li

**Affiliations:** 1 Department of Gastrointestinal and Gland Surgery, The First Affiliated Hospital of Guangxi Medical University, No. 6 Shuangyong Road, Guangxi18 Zhuang Autonomous Region, Nanning, 530021 P.R. China; 2 Department of Thyroid Surgery, the Fourth Affiliated Hospital of Guangxi Medical University, Liuzhou, 545027 China

**Keywords:** Papillary thyroid carcinoma, Central lymph nodes, Lymph nodes posterior to the right recurrent laryngeal nerve, Metastasis prediction, Nomogram

## Abstract

**Background:**

Metastasis to lymph nodes posterior to the right recurrent laryngeal nerve (LN-prRLN) in papillary thyroid carcinoma (PTC) presents significant surgical challenges due to its deep anatomical location and association with disease recurrence.

**Objective:**

To identify risk factors for LN-prRLN metastasis and develop a validated prediction model for clinical decision-making.

**Methods:**

A retrospective analysis of 341 PTC patients underwent LN-prRLN dissection (May 2022–September 2024) at the First Affiliated Hospital of Guangxi Medical University was conducted. Clinicopathological characteristics were compared between metastasis-positive and negative groups. Independent risk factors were identified through univariate and multivariate logistic regression and utilized to construct a nomogram. Model performance was assessed using Receiver operating characteristic (ROC) curves, calibration plots, and decision curve analysis (DCA).

**Results:**

Male (odds ratio = 2.670, 95%CI:1.094–6.516), Tumor diameter (odds ratio = 1.931, 95%CI:1.140–3.270), Multifocality (odds ratio = 3.658, 95%CI:1.251–10.692), LN-arRLN metastasis (odds ratio = 1.340, 95%CI:1.122–1.602), Lateral lymph node metastasis (odds ratio = 7.815, 95%CI:2.857–21.379), Extrathyroidal extension (OR = 3.627, 95%CI:1.133–11.611) were identified as the independent risk factors for LN-prRLN metastasis. The nomogram demonstrated excellent discrimination (AUC: training cohort = 0.889; validation cohort = 0.858). The calibration curves demonstrated good concordance between predicted probabilities and the actual observed probabilities. The DCA curve indicates robust clinical utility for the model.

**Conclusion:**

This study extends prior research by identifying additional independent risk factors, the model evaluation results indicated satisfactory predictive performance, facilitates individualized surgical planning to balance therapeutic efficacy against procedural risks.

## Introduction

Thyroid carcinoma represents the most prevalent endocrine malignancy worldwide, with its incidence exhibiting a dramatic upward trajectory over the past decade. In China, this burden is particularly significant: 466,100 new cases occurred in 2022, accounting for 9.7% of all malignancies and ranking third in national cancer incidence. Notably, epidemiological projections suggest an alarming annual growth rate of 20% [1, 2]. Papillary thyroid carcinoma (PTC) constitutes 80% of cases and demonstrates 10-year survival exceeding 90% [3]. However, it is prone to central lymph nodes metastasis (CLNM) in the early stage, which occurs in 30–90% of patients [4]. CLNM will increase the recurrence and mortality [5, 6], so complete lymph node dissection is the basis for ensuring the effect of surgical treatment of thyroid cancer.

Anatomically, the right central compartment warrants special consideration. Unlike the left side, where the esophagus lies directly posterior to the trachea, the right tracheoesophageal groove creates a potential space posterior to the right recurrent laryngeal nerve (RLN). This distinct anatomy facilitates the existence of lymph nodes posterior to the right RLN (LN-prRLN) and lymph nodes anterior to the right RLN(LN-arRLN) [7]. LN-prRLN’s deep location (bounded superiorly by the tracheal-nerve junction, inferiorly by the innominate artery/pleural roof, laterally by the carotid sheath, and medially by the esophagus) limits preoperative detection by ultrasound or computed tomography (CT). The clinical dilemma persists: complete dissection of LN-prRLN may reduce recurrence but risks RLN injury and hypoparathyroidism, whereas omitting dissection risks residual disease. Current guidelines lack explicit recommendations regarding LN-prRLN management, creating substantial practice variation [8].

Previous efforts to identify LN-prRLN metastasis predictors have yielded inconsistent results, with studies limited by single-center designs, small samples, or inadequate validation. Moreover, no clinically implemented prediction model exists to stratify metastasis risk and guide personalized dissection strategies. This study aimed to identify independent clinicopathological risk factors for LN-prRLN metastasis, develop and validate a nomogram prediction model integrating these factors, and provide evidence-based tools for intraoperative decision-making.

## Materials and methods

### Patients

We retrospectively analyzed 341 PTC patients underwent LN-arRLN and LN-prRLN dissection between May 2022 and September 2024 at the First Affiliated Hospital of Guangxi Medical University. All surgical procedures were performed by a same specialized surgical team.

Inclusion criteria:

- (1) Complete clinicopathological data;
- (2) Primary surgery;
- (3) Histologically confirmed PTC.
- Exclusion criteria:
- (1) Incomplete clinical records;
- (2) Coexistence with other head and neck malignancies.
- All patients were randomly divided into training (*n* = 239, 70%) and validation (*n* = 102, 30%) cohorts. The training cohort was used to construct the model, and the validation cohort was used to validate the model.

### Clinicopathological data

The clinicopathological data collected in this study included: gender, age, thyroid-stimulating hormone (TSH) levels, tumor location, tumor diameter, presence of Hashimoto’s thyroiditis (HT), multifocality, extrathyroidal extension (ETE), lymph node metastasis in LN-arRLN, LN-prRLN, and lateral lymph node.

### Statistical analyses

IBM SPSS Statistics 27.0 and RStudio 4.2.2 were used for analyses. Continuous variables were reported as mean ± SD or median [Q1, Q3], compared using t-tests or Mann-Whitney U tests. Categorical variables were analyzed by χ² or Fisher’s exact tests. Independent risk factors were identified via binary logistic regression (*P* < 0.05). A nomogram was developed and validated using ROC curves, calibration plots, and DCA.

## Results

### Comparison of basic clinicopathological characteristics of patients in the training cohort and validation cohort

According to predefined inclusion and exclusion criteria, 341 patients were enrolled, including 77 male and 264 female patients (mean age 39.4 ± 11.7 years). Of these, 107 patients were being 45 years or older and 234 were younger than 45 years old. Postoperative histopathological assessment revealed lymph node metastasis rates of 54.8% (187/341) for the LN-arRLN and 30.8% (105/341) for the LN-prRLN. All patients were assigned to either a training cohort (*n* = 239) or a validation cohort (*n* = 102) in a 7:3 ratio using a random number sequence. Table 1 presents the comparison of baseline clinicopathological characteristics between the training and validation cohorts. LN-prRLN metastasis was observed in 71 patients (29.7%) in the training cohort and in 34 patients (33%) in the validation cohort. No significant differences (all *P* > 0.05) were observed between the cohorts in baseline characteristics.

**Table 1:** Comparison of basic clinicopathological characteristics between training cohort and validation cohort

basic clinicopathological characteristics	Training cohort *n* = 239	validation cohort *n* = 102	*P*-value
Gender [n (%)]			> 0.05
Male	48(20.0%)	29(28.4%)	
Female	191(80.0%)	73(71.6%)	
Age, y ± SD	39.2 ± 11.8	40.1 ± 11.9	> 0.05
Tumor diameter [median (Q1, Q3)], cm	0.9[0.6,1.4]	0.95[0.7,1.4]	> 0.05
TSH [median (Q1, Q3)],µIU/mL	1.64[1.10,2.32]	1.59[0.96,2.46]	> 0.05
Multifocality [n (%)]			>0.05
Present	36(15.1%)	14(13.7%)	
Absent	203(84.9%)	88(86.3%)	
Location of the tumor [n (%)]			> 0.05
Upper pole	59(24.7%)	30(29.4%)	
Middle pole	119(49.8%)	46(45.1%)	
Lower pole	61(25.5%)	26(25.5%)	
Number of LN-arRLN metastases [median (Q1, Q3)], n	1.0[0,3.0]	1.0[0,4.0]	> 0.05
LN-prRLN metastases [n (%)]	71(29.7%)	34(33.3%)	> 0.05
	168(70.3%)	68(66.7%)	
Extrathyroidal extension [n (%)]			> 0.05
Present	27(11.3%)	10(9.8%)	
Absent	212(88.7%)	92(90.2%)	
Lateral lymph node metastasis [n (%)]			> 0.05
Present	44(18.4%)	20(19.6%)	
Absent	195(81.6%)	82(80.4%)	
Hashimoto’s thyroiditis [n (%)]			> 0.05
Present	75(31.4%)	29(28.4%)	
Absent	164(68.6%)	73(71.6%)	

### Univariate analysis of clinicopathological characteristics training cohort

Patients in the training cohort were categorized into LN-prRLN-positive and LN-prRLN-negative groups based on metastasis status, and the differences in clinicopathological characteristics were compared (Table 2). Univariate analysis identified significant associations with LN-prRLN metastasis for: gender (*P* < 0.01), age younger than 45 years old(*P* < 0.05), tumor diameter(*P* < 0.01), multifocality (*P* < 0.01), LN-arRLN metastasis (*P* < 0.01), ETE (*P* < 0.01) and LLNM (*P* < 0.01).

**Table 2:** Univariate analysis of clinicopathological characteristics training cohort

clinicopathological characteristics	LN-prRLN-positive *n* = 71	LN-prRLN-negative *n* = 168	*P*-value
Gender [n (%)]			<0.01
Male	23(36.2%)	25(22.2%)	
Female	48(63.8%)	143(77.8%)	
Age, [years, n(%)]			<0.05
≥ 45	15(21.1%)	59(38.1%)	
<45	56(78.9%)	109(61.9%)	
Tumor diameter [median (Q1, Q3)], cm	1.3[0.9,2.6]	0.6[0.8,1.1]	<0.01
TSH [median (Q1, Q3)], µIU/mL	1.77[1.06,2.34]	1.57[1.12,2.28]	0.547
Multifocality			
Present	19(26.8%)	17(10.1%)	<0.01
Absent	52(73.2%)	151(89.9%)	
Location of the tumor [n (%)]			0.454
Upper pole	16(22.5%)	43(25.6%)	
Middle pole	39(54.0%)	80(47.6%)	
Lower pole	16(22.5%)	45(26.8%)	
Number of LN-arRLN metastases [median (Q1, Q3)], n	5[2,5]	0[0,1]	<0.01
Extrathyroidal extension			<0.01
Present	16(22.5%)	11(6.5%)	
vAbsent	55(77.5%)	157(93.5%)	
Lateral lymph node metastasis [n (%)]			<0.01
Present	35(49.3%)	9(5.4%)	
Absent	36(50.7%)	159(94.6%)	
Hashimoto’s thyroiditis [n (%)]			0.109
Present	17(23.9%)	58(34.5%)	
Absent	54(76.1%)	110(65.5%)	

These factors were subsequently analyzed using binary logistic regression (Table 3). Multivariate analysis revealed the following independent risk factors for LN-prRLN metastasis: male gender (*P* < 0.05), larger tumor diameter (*P* < 0.05), multifocality (*P* < 0.05), LN-arRLN metastasis (*P* < 0.05), LLNM (*P* < 0.01), and ETE (*P* < 0.05). To assess potential multicollinearity among the defined independent risk factors, we performed collinearity analysis (Table 4). All variance inflation factor (VIF) values were less than 5, indicating no significant multicollinearity among the included variables.

**Table 3:** Multivariate analysis of LN-prRLN metastasis

Risk factor	Odds ratio (95%CI)	*P*-value
Gender (male)	2.670(1.094–6.516)	<0.05
Age	0.988(0.953–1.023)	0.395
Tumor diameter	1.931(1.140–3.270)	< 0.05
Multifocality	3.658(1.251–10.692)	<0.05
LN-arRLN metastases	1.340(1.122–1.602)	<0.05
Lateral lymph node metastasis	7.815(2.857–21.379)	< 0.01
Extrathyroidal extension	3.627(1.133–11.611)	<0.05

**Table 4:** Collinearity analysis of independent risk factors

Risk factor	Variance inflation factor (VIF) values	Tolerance
Gender (male)	1.062	0.942
Tumor diameter	1.373	0.728
Multifocality	1.065	0.939
LN-arRLN metastases	1.613	0.620
Lateral lymph node metastasis	1.388	0.721
Extrathyroidal extension	1.056	0.947

### Construction of prediction model

Based on the six independent risk factors identified by multivariate analysis, we constructed a nomogram to predict LN-prRLN metastasis in PTC patients. Within the nomogram, the length of each variable’s line segment corresponds to its relative contribution to metastasis risk. The top axis indicates the points assigned to each variable value, while the bottom axes display the total points and their corresponding predicted probability of LN-prRLN metastasis.

**Fig. 1:**
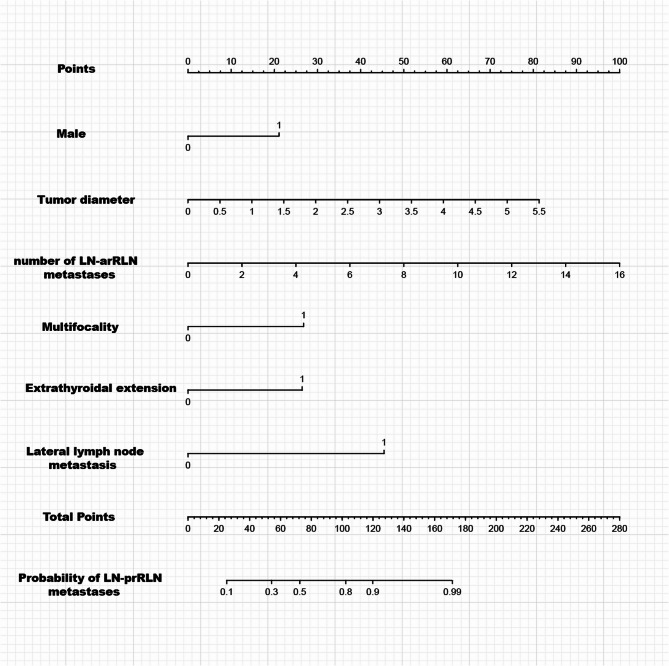
Nomogram predicting LN-prRLN metastasis risk. *Links to the online version:https://nomogram-6215816.shinyapps.io/LN-prRLN/

The clinical application of the nomogram is demonstrated in the following illustrative case: A male patient presented with multifocal right-lobe tumors (largest tumor diameter: 1.5 cm), no abnormal lateral lymph node on preoperative ultrasound, and no intraoperative evidence of extrathyroidal extension. Frozen section analysis identified metastatic involvement in three LN-arRLN. Inputting these variables into the nomogram yielded an estimated total score of 60, corresponding to a 36% predicted probability of LN-prRLN metastasis. Furthermore, we provide an interactive online version to facilitate rapid risk assessment (Fig. 1).

### Validation and evaluation of prediction model

**Fig. 2:**
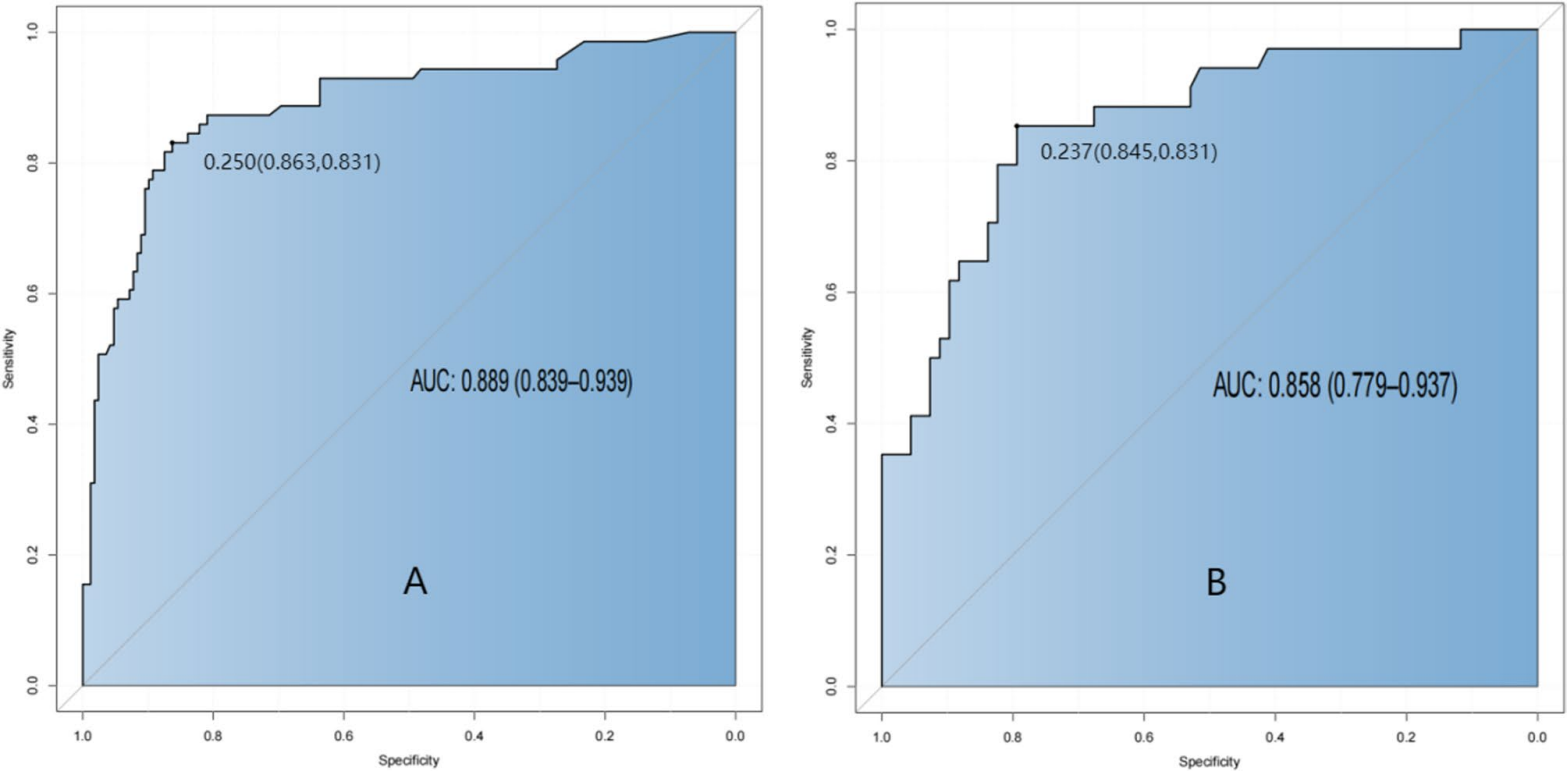
ROC curves of training cohort and validation cohort. *A ROC curves of the training cohort; **B**: ROC curves of the verification cohort

The discriminative ability of a prediction model—its capacity to correctly distinguish between individuals who will and will not experience the outcome event—was evaluated using ROC curves. ROC curves plot the sensitivity against the specificity across various classification thresholds. The AUC Quantifies overall discriminative performance, with higher AUC values indicating superior discrimination. In this model, the AUC was 0.889 (95% CI, 0.839–0.939) for the training cohort and 0.858 (95% CI, 0.779–0.937) for the validation cohort (Fig. 2). At the optimal cut-off defined by the maximum Youden index, sensitivity and specificity were 83.1% and 86.3%, respectively, with corresponding positive and negative predictive values of 80.8% and 84.5%. These results indicate robust discriminative ability of the model.

**Fig. 3:**
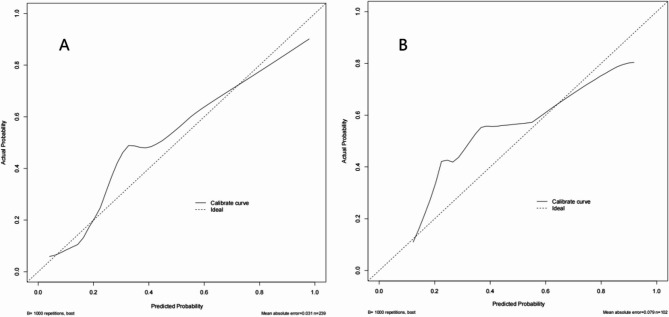
Calibration curves of training cohort and validation cohort. *A Calibration curves for the training cohort; **B**: Calibration curves for the validation cohort

Calibration assesses the agreement between predicted and observed outcome risks. For internal validation, we generated a calibration curve (Fig. 3) using 1000 bootstrap resamples, comparing model-predicted probabilities of LN-prRLN metastasis with actual outcomes. The x-axis represents predicted probability, the y-axis represents observed frequency, and the diagonal dashed line indicates perfect calibration (predicted = observed). The calibration curves for both training and validation cohorts closely aligned with the ideal Line, and the average absolute error of the training and validation cohorts was 0.031 and 0.079, demonstrating excellent agreement between model predictions and actual probabilities.

**Fig. 4:**
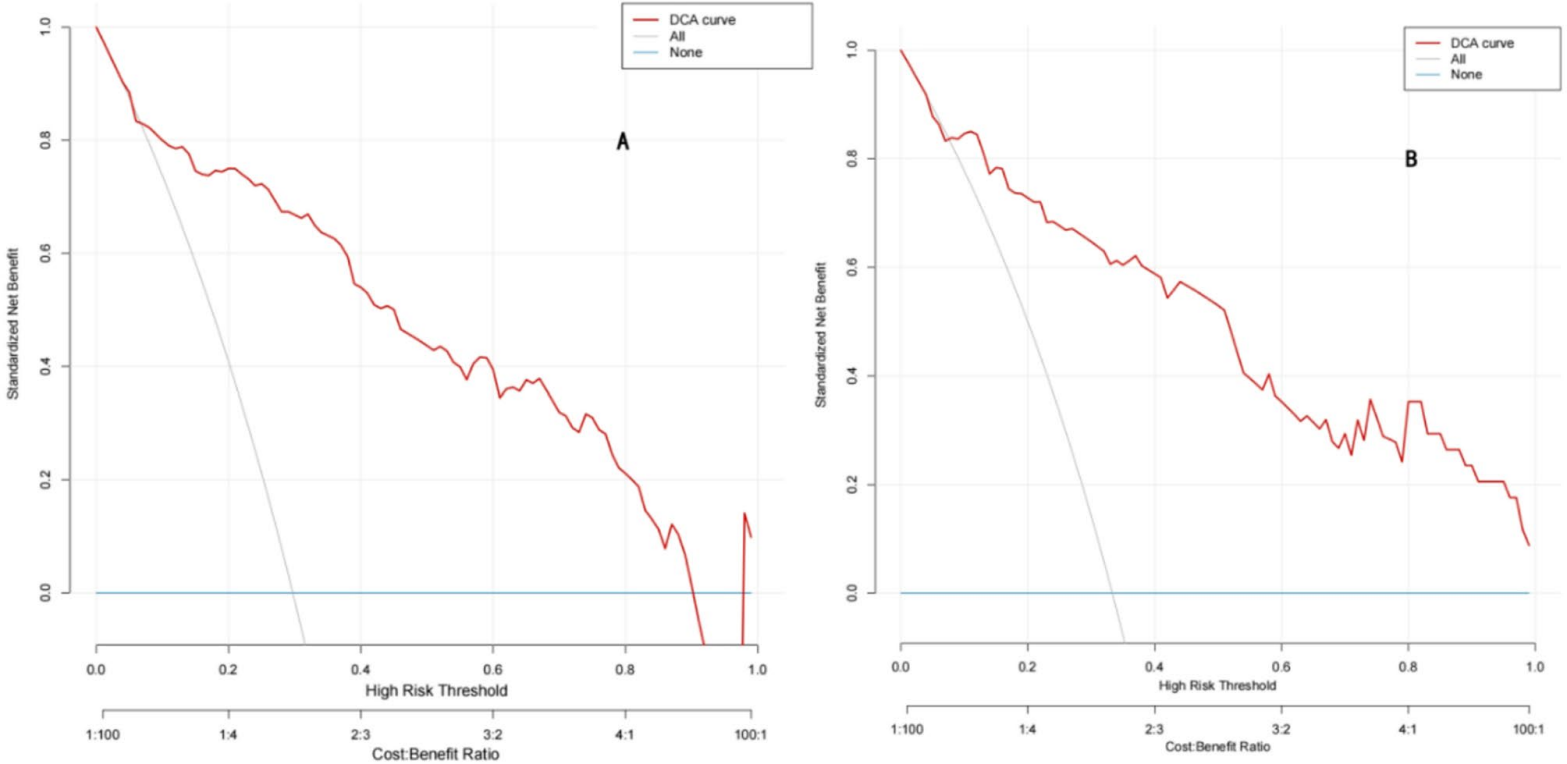
DCA curves of training cohort and validation cohort. *A DCA curves for the training cohort; **B**: DCA curves for the validation cohort

Clinical decision curve analysis (DCA) is a widely used tool for evaluating the clinical utility of prediction models. A key advantage of DCA over ROC analysis is its integration of patient or decision-maker preferences. The DCA curve plots net benefit (benefit of treating true-positive patients minus the harms of unnecessary dissection in LN-prRLN-negative patients, including the consequences of missed dissection.) on the y-axis against threshold probability on the x-axis. The threshold probability represents the minimum predicted probability of LN-prRLN at which a decision to perform LN-prRLN dissection is recommended. The gray curve represents LN-prRLN dissection was performed for all patients. Under this scenario, the model’s net benefit progressively declines as the threshold probability increases. The blue curve represents the scenario where LN-prRLN dissection was not performed in any patient, where the model’s net benefit remains at zero. In this study, the DCA curve (Fig. 4) demonstrated superior net benefit compared to the strategies of performing LN-prRLN for all patients or for no patients. This indicates robust clinical utility for the model within this clinically relevant risk threshold range.

## Discussion

PTC represents the most common histological subtype of thyroid cancer and is associated with a generally favourable prognosis. However, due to its high propensity for CLNM and strong correlation with recurrence, identifying optimal management strategies for LN-prRLN represents a critical need. In this study, LN-prRLN exhibited a metastasis rate of 30.3%. This finding aligns with reported rates across multiple studies, which range from approximately 9–30% [9–11]. Although the LN-prRLN metastasis rate is lower than the overall CLNM rate, Clayman et al. reported that 59.0% of recurrent central lymph nodes identified during reoperation were located posterior and dorsal to the right RLN (i.e., LN-prRLN), significantly more frequent than in other central subcompartments [12]. Such recurrences often necessitate hazardous reoperations within scar tissue. Consequently, LN-prRLN metastasis may be a primary contributor to postoperative recurrence.

Previous studies have identified patients younger than 45 years old, male, larger tumor diameter, multifocality, ETE, metastasis to the LN-arRLN, and LLNM as independent risk factors for LN-prRLN [13, 14]. Our findings corroborate these risk factors. Age was not identified as an independent risk factor in this study. While a previous study have reported similar findings, this variation may be attributed to differences in the study populations [7].

While females exhibit a higher incidence of PTC, studies indicate that male patients frequently present with more aggressive clinicopathological features [15]. The relationship between gender and lymph node metastasis risk may also involve hormonal influences [16, 17]. The LN-prRLN metastasis rate in our cohort was significantly higher in male patients (51.9%, 40/77) than in females (24.6%, 65/264). This result is consistent with the previous statement. In the study by Yang et al., gender was not identified as an independent risk factor. Interestingly, tumor location in the lower portion of the thyroid was found to be an independent risk factor for LN-prRLN in their cohort, whereas in our study, tumor location demonstrated no significant association, with tumors in the middle portion being more frequently(54.0%) associated with LN-prRLN occurrence [18].

Based on ROC curve analysis maximizing the Youden index, we identified 1 cm as the optimal threshold for predicting LN-prRLN metastasis. The metastasis rate was significantly higher in tumours > 1 cm (51.0%) compared to those ≤ 1 cm (15.1%). This finding aligns with Zhang et al., who identified tumour size > 1 cm as an independent risk factor for LN-prRLN metastasis [7], and is consistent with Shan et al., who reported an optimal cut-off of 1.27 cm [19]. In contrast, Ito et al. proposed ≥ 2 cm as an independent risk factor [20]. Taken together, these data indicate a substantially lower LN-prRLN metastasis rate in microcarcinomas (≤ 1 cm). Consequently, preoperative ultrasound indicating a tumour exceeding 1 cm warrants meticulous intraoperative exploration of the tissue posterior to the right recurrent laryngeal nerve.

We defined multifocality as the presence of two or more distinct cancer foci within the right lobe. Our analysis revealed that multifocality increased the risk of LN-prRLN metastasis by 3.7-fold. In previous prediction models [13, 14], multifocality is not incorporated as a predictor. This discrepancy may be caused by variations in the definition of multifocality across studies. While multifocality incidence may be independent of tumor size, the biological consequence of multifocality functionally equates to an increase in total tumor burden. Consequently, multifocality correlates with higher rates of ETE, lymph node metastasis, and recurrence [21]. Thus, routine LN-prRLN dissection is recommended for patients with multifocal disease in the right lobe.

ETE represents a hallmark of aggressive thyroid cancer behavior and is a significant prognostic factor. The presence of ETE substantially elevates the risk of tumor recurrence and mortality [22]. Corroborating previous reports, our study found a high LN-prRLN metastasis rate (65.4%) in patients with ETE. Zhou et al. identified ETE as the most critical predictor for LN-prRLN metastasis (rate: 53.8%) [9]. Furthermore, Seifert et al. found that even minimal ETE (microscopic extension identified pathologically) significantly increased lymph node metastasis rates (61.4% vs. 28.5% without minimal ETE) [23]. Preoperative ultrasonography remains the primary modality for ETE assessment, with suggestive features including tumor proximity to the thyroid capsule, capsular contour bulging, and loss of the capsular echogenic line. Hence, evidence of ETE, either preoperatively or intraoperatively, necessitates careful evaluation and consideration of LN-prRLN dissection.

CLNM is a well-established predictor of recurrence and mortality in thyroid cancer [24, 25]. Our data demonstrate a strong correlation between the number of metastatic LN-arRLN and the risk of LN-prRLN metastasis. Specifically, Shan et al. reported an 8.4-fold increased risk of LN-prRLN metastasis when ≥ 2 LN-arRLN are involved [19]. Therefore, for patients with suspected metastasis to the LN-prRLN, resection of suspicious LN-arRLN with intraoperative frozen section analysis should be considered. This approach substantially facilitates the intraoperative assessment of LN-prRLN. Additionally, Xiao et al. identified left-sided CLNM as an independent risk factor for LN-prRLN metastasis (rate: 42.42% in patients with left CLNM) [26], potentially reflecting the heightened aggressiveness associated with bilateral central neck involvement.

In conclusion, this study extends prior research by identifying additional independent risk factors. To facilitate clinical application, we developed a nomogram to predict LN-prRLN metastasis. This model enables surgeons to objectively assess individual patient risk, thereby informing decisions regarding both standardized and individualized treatment strategies. However, Limitations should be acknowledged: 1. Lack of genetic variables: Preoperative genetic testing (e.g., for BRAF V600E or TERT promoter mutations) was not routinely available for inclusion in the model. Future work will implement preoperative genetic testing and integrate genomic data to refine predictive accuracy. 2.Sample scope: While the model was developed using a single-center cohort, the relative scarcity of patients undergoing LN-prRLN dissection necessitates further validation in larger, multi-center cohorts to ensure broad generalizability.

The original data supporting the conclusions of this article will be made available by the authors.

## Data Availability

The original data supporting the conclusions of this article will be made available by the authors.

## References

[1] Han B, Zheng R, Zeng H, et al. Cancer incidence and mortality in China, 2022. J Natl Cancer Center. 2024;4(1):47–53. 39036382 10.1016/j.jncc.2024.01.006 PMC11256708

[2] Xia C, Dong X, Li H, et al. Cancer statistics in China and United States, 2022: profiles, trends, and determinants. Chin Med J (Engl). 2022;135(5):584–90. 35143424 10.1097/CM9.0000000000002108 PMC8920425

[3] Pamedytyte D, Simanaviciene V, Dauksiene D, et al. Association of microrna expression and BRAF(V600E) mutation with recurrence of thyroid cancer. Biomolecules. 2020;10(4):625. 32316638 10.3390/biom10040625 PMC7226510

[4] Qu H, Sun GR, Liu Y, et al. Clinical risk factors for central lymph node metastasis in papillary thyroid carcinoma: a systematic review and meta-analysis. Clin Endocrinol (Oxf). 2015;83(1):124–32. 25130203 10.1111/cen.12583

[5] Jang SW, Park JH, Kim HR, et al. Recurrence risk evaluation in patients with papillary thyroid carcinoma: multicenter machine learning evaluation of lymph node variables. Cancers (Basel). 2023;15(2):550. 10.3390/cancers15020550 PMC9856505 36672498

[6] Podnos YD, Smith D, Wagman LD, et al. The implication of lymph node metastasis on survival in patients with well-differentiated thyroid cancer. Am Surg. 2005;71(9):731–4. 16468507 10.1177/000313480507100907

[7] Zhang PY, Zhang B,Bu JL et al. Prospective analysis of the risk factors and clinical indications of dissection of lymph node posterior to right recurrent laryngeal nerve in 283 cases of papillary thyroid carcinoma [J]. Chin J Oncol 2014,36(2):109–14(in Chinese). 24796458

[8] Haugen BR, Alexander EK, Bible KC, et al. 2015 American thyroid association management guidelines for adult patients with thyroid nodules and differentiated thyroid cancer. Thyroid. 2016;26(1):1–133. 26462967 10.1089/thy.2015.0020 PMC4739132

[9] Zhou M, Duan Y, Ye B, et al. Pattern and predictive factors of metastasis in lymph nodes posterior to the right recurrent laryngeal nerve in papillary thyroid carcinoma. Front Endocrinol (Lausanne). 2022;13:914946. 35923627 10.3389/fendo.2022.914946 PMC9339603

[10] Li C, Xiang J, Wang Y. Risk factors for predicting lymph nodes posterior to right recurrent laryngeal nerve (LN-prRLN) metastasis in thyroid papillary carcinoma: a meta-analysis. Int J Endocrinol. 2019;2019:7064328. 31049063 10.1155/2019/7064328 PMC6462345

[11] Park YM, Lee SM, Kim DW, et al. Predictive factors of right paraesophageal lymph node metastasis in papillary thyroid carcinoma: single center experience and meta-analysis. PLoS ONE. 2017;12(5):e0177956. 28545107 10.1371/journal.pone.0177956 PMC5435339

[12] Clayman GL, Agarwal G, Edeiken BS, et al. Long-term outcome of comprehensive central compartment dissection in patients with recurrent/persistent papillary thyroid carcinoma. Thyroid. 2011;21(12):1309–16. 22136266 10.1089/thy.2011.0170 PMC3968954

[13] Li J, He G, Tong Y, et al. A novel scoring system for the risk of papillary thyroid cancer metastases in lymph nodes posterior to the right of the recurrent laryngeal nerve. Endocr Pract. 2021;27(1):15–20. 33471728 10.4158/EP-2020-0129

[14] Luo Y, Xu XC, Shen J, et al. Model of lymph node metastasis posterior to the right recurrent laryngeal nerve in papillary thyroid carcinoma. Cancer Manag Res. 2018;10:2449–55. 30122994 10.2147/CMAR.S167997 PMC6084087

[15] Kim H, Kwon H, Moon BI. Predictors of recurrence in patients with papillary thyroid carcinoma: does male sex matter? Cancers (Basel). 2022;14(8):1896. 35454803 10.3390/cancers14081896 PMC9030936

[16] Shi P, Yang D, Liu Y, et al. A protective factor against lymph node metastasis of papillary thyroid cancer: female gender. Auris Nasus Larynx. 2023;50(3):440–9. 36253315 10.1016/j.anl.2022.10.001

[17] Rahbari R, Zhang L, Kebebew E. Thyroid cancer gender disparity. Future Oncol. 2010;6(11):1771–9. 21142662 10.2217/fon.10.127 PMC3077966

[18] Gong Y, Zuo Z, Tang K, Xu Y, Zhang R, Peng Q, et al. Multimodal predictive factors of metastasis in lymph nodes posterior to the right recurrent laryngeal nerve in papillary thyroid carcinoma. Front Endocrinol (Lausanne). 2023;14:1187825. 37501788 10.3389/fendo.2023.1187825 PMC10369781

[19] Shan Sw, Liu CB, Wei W, et al. Evaluation of the clinical and ultrasound characteristics in the metastasis of lymph node posterior to right recurrent laryngeal nerve in papillary thyroid carcinoma[J]. J Xuzhou Med Univ. 2021;41(3):200–4. (in Chinese).

[20] Ito Y, Fukushima M, Higashiyama T, et al. Incidence and predictors of right paraesophageal lymph node metastasis of N0 papillary thyroid carcinoma located in the right lobe. Endocr J. 2013;60(3):389–92. 23182918 10.1507/endocrj.ej12-0362

[21] Qu N, Zhang L, Ji QH, et al. Number of tumor foci predicts prognosis in papillary thyroid cancer. BMC Cancer. 2014;14:914. 25471041 10.1186/1471-2407-14-914 PMC4289292

[22] Lin JD, Hsueh C, Chao TC. Soft tissue invasion of papillary thyroid carcinoma. Clin Exp Metastasis. 2016;33(6):601–8. 27154220 10.1007/s10585-016-9800-3 PMC4947096

[23] Seifert R, Schäfers MA, Heitplatz B, et al. Minimal extrathyroid extension in papillary micro carcinoma of the thyroid is an independent risk factor for relapse through lymph node and distant metastases[J]. J Nucl Med. 2021;62(12):1702–9. 33771902 10.2967/jnumed.121.261898 PMC8612207

[24] Zaydfudim V, Feurer ID, Griffin MR et al. The impact of lymph node involvement on survival in patients with papillary and follicular thyroid carcinoma[J]. Surgery, 2008, 144(6): 1070-7; discussion 1077-8. 10.1016/j.surg.2008.08.034 19041020

[25] Pelizzo MR, Boschin IM, Toniato A, et al. Papillary thyroid carcinoma: 35-year outcome and prognostic factors in 1858 patients. Clin Nucl Med. 2007;32(6):440–4. 17515749 10.1097/RLU.0b013e31805375ca

[26] Xiao X, Wu Y, Zou L, et al. Value of dissection of lymph nodes posterior to the right recurrent laryngeal nerve in patients with cN(0) papillary thyroid carcinoma. Gland Surg. 2022;11(7):1204–11. 35935559 10.21037/gs-22-337 PMC9346216

